# Searching for the definition of macrosomia through an outcome-based approach in low- and middle-income countries: a secondary analysis of the WHO Global Survey in Africa, Asia and Latin America

**DOI:** 10.1186/s12884-015-0765-z

**Published:** 2015-12-03

**Authors:** Jiangfeng Ye, Maria Regina Torloni, Erika Ota, Kapila Jayaratne, Cynthia Pileggi-Castro, Eduardo Ortiz-Panozo, Pisake Lumbiganon, Naho Morisaki, Malinee Laopaiboon, Rintaro Mori, Özge Tunçalp, Fang Fang, Hongping Yu, João Paulo Souza, Joshua Peter Vogel, Jun Zhang

**Affiliations:** Ministry of Education–Shanghai Key Laboratory of Children’s Environmental Health, Xinhua Hospital, Shanghai Jiao Tong University School of Medicine, Shanghai, China; UNDP • UNFPA • UNICEF • WHO • World Bank Special Programme of Research, Development and Research Training in Human Reproduction, Department of Reproductive Health and Research, World Health Organization, Geneva, Switzerland; Department of Internal Medicine, Post Graduate program of Evidence Based Healthcare, São Paulo Federal University, São Paulo, SP Brazil; Department of Health Policy, National Center for Child Health and Development, Tokyo, Japan; Family Health Bureau, Ministry of Health, 231, De Saram Place, Colombo 10, Sri Lanka; Department of Pediatrics, Ribeirão Preto Medical School, University of São Paulo, Ribeirão Preto, SP Brazil; Center for Population Health Research, National Institute of Public Health, Cuernavaca, Mexico; Department of Obstetrics & Gynaecology, Faculty of Medicine, Khon Kaen University, Khon Kaen, Thailand; Division of Lifecourse Epidemiology, Department of Social Medicine, National Center for Child Health and Development, Tokyo, Japan; Department of Biostatistics & Demography, Faculty of Public Health, Khon Kaen University, Khon Kaen, Thailand; School of Public Health, Guilin Medical College, Guangxi, China; Department of Social Medicine, Ribeirão Preto Medical School, University of São Paulo, Ribeirão Preto, SP Brazil

**Keywords:** Macrosomia, Maternal mortality, Maternal morbidity, Neonatal mortality, Neonatal morbidity

## Abstract

**Background:**

No consensus definition of macrosomia currently exists among researchers and obstetricians. We aimed to identify a definition of macrosomia that is more predictive of maternal and perinatal mortality and morbidity in low- and middle-income countries.

**Methods:**

We conducted a secondary data analysis using WHO Global Survey on Maternal and Perinatal Health data on Africa and Latin America from 2004 to 2005 and Asia from 2007 to 2008. We compared adverse outcomes, which were assessed by the composite maternal mortality and morbidity index (MMMI) and perinatal mortality and morbidity index (PMMI) in subgroups with birthweight (3000–3499 g [reference group], 3500–3999 g, 4000–4099 g, 4100–4199 g, 4200–4299 g, 4300–4399 g, 4400–4499 g, 4500–4999 g) or country-specific birthweight percentile for gestational age (50^th^–74^th^ percentile [reference group], 75^th^–89^th^, 90^th^–94^th^, 95^th^–96^th^, and ≥97^th^ percentile). Two-level logistic regression models were used to estimate odds ratios of MMMI and PMMI.

**Results:**

A total of 246,659 singleton term births from 363 facilities in 23 low- and middle-income countries were included. Adjusted odds ratios (aORs) for intrapartum caesarean sections exceeded 2.0 when birthweight was greater than 4000 g (2 · 00 [95 % CI: 1 · 68, 2 · 39], 2 · 42 [95 % CI: 2 · 02, 2 · 89], 2 · 01 [95 % CI: 1 · 74, 2 · 33] in Africa, Asia and Latin America, respectively). aORs of MMMI reached 2.0 when birthweight was greater than 4000 g, 4500 g in Asia and Africa, respectively. aORs of PMMI approached to 2.0 (1 · 78 [95 % CI: 1 · 16, 2 · 74]) when birthweight was greater than 4500 g in Latin America. When birthweight was at the 90^th^ percentile or higher, aORs of MMMI and PMMI increased, but none exceeded 2.0.

**Conclusions:**

The population-specific definition of macrosomia using birthweight cut-off points irrespective of gestational age (4500 g in Africa and Latin America, 4000 g in Asia) is more predictive of maternal and perinatal adverse outcomes, and simpler to apply compared to the definition based on birthweight percentile for a given gestational age.

**Electronic supplementary material:**

The online version of this article (doi:10.1186/s12884-015-0765-z) contains supplementary material, which is available to authorized users.

## Background

“Macrosomia” is a term that describes a very large fetus or neonate. The condition may be caused by constitutional/genetic factors, maternal obesity and/or excessive gestational weight gain, or maternal hyperglycemia due to pre-existing diabetes or gestational diabetes that were not adequately controlled [1]. In low-and middle- income countries (LMICs) or settings where antenatal care is sub–optimal, poorly controlled diabetes or undiagnosed gestational diabetes may be a more important cause for macrosomia than in high-income countries, where antenatal care is better. In high-income countries, the prevalence of macrosomia has been increasing in the last two to three decades [2, 3]. But in many LMICs, macrosomia is still not perceived to have the same priority as other public health problems (e.g., HIV) [4]. However, with the increasing prevalence of maternal obesity and diabetes [5, 6] a parallel increase in macrosomic infants might be expected in LMICs. Complicated deliveries related to macrosomia could lead to more severe adverse outcomes in resource-poor settings due to limited availability of obstetric care. Thus, a precise definition of macrosomia that is more predictive of maternal and perinatal mortality and morbidity is needed. In this study, we aimed to explore a definition through an outcome-based approach and comparing commonly used definitions currently.

Currently, no consensus definition exists among researchers and obstetricians. The most commonly used definition is based on birthweight cut-off points (e.g., 4000 g or 4500 g) [3, 7–9]. As it is increasingly recognized that racial variation in birthweight is substantial, more and more studies are using specific birthweight percentiles as cut-off points at a given gestational week (e.g., P_90_ or P_97_) based on the concept of large-for-gestational-age (LGA) [2, 10]. Furthermore, most studies on macrosomia have focused on Caucasian populations in high-income countries, and very few studies on the topic focus on LMICs [2].

In this study we analyzed data from 23 LMICs in Africa, Asia, and Latin America that participated in the World Health Organization (WHO) Global Survey on Maternal and Perinatal Health (2004–2008). We assessed commonly used definitions of the term ‘macrosomia’ through an outcome-based approach. Two types of definitions were compared: one based on empirical absolute birthweight and the other on the country-specific birthweight percentile at each gestational week. We aimed to identify a definition that was more predictive of maternal and perinatal mortality and morbidity in term pregnancies in LMICs, which also takes into account regional variation.

## Methods

### Study design and data extraction

The general objective of the WHO Global Survey on Maternal and Perinatal Health was to create a global database on health services and outcomes for maternal and perinatal health, which concentrated on the relationship between mode of delivery and perinatal outcomes [2, 11]. This survey has previously been described in detail elsewhere [2, 11, 12]. A total of 373 facilities in 24 countries in Africa, Asia and Latin America participated in this survey. Data collection was carried out in 2004–05 in Africa and Latin America, and in 2007–08 in Asia. Trained data collectors extracted data from medical records and completed standardized forms. Gestational age was calculated based on the difference between the estimated and actual delivery date in the medical records. Data related to outcomes were obtained until discharge from the hospital. Maternal weight was defined differently as described previously: in Africa and Latin America, maternal weight was the weight recorded at the first antenatal care visit, while in Asia it was the weight at the last visit before delivery [2, 11].

This is a secondary data analysis using data from the WHO Global Survey on Maternal and Perinatal Health. The protocol of this survey was approved by the ethics committees at the WHO and in all participating centres [11]. We obtained permission to use this data from Department of Reproductive Health and Research, WHO. An individual informed consent was not obtained because in this survey data were extracted from medical records without individual identification [11].

**Fig. 1:**
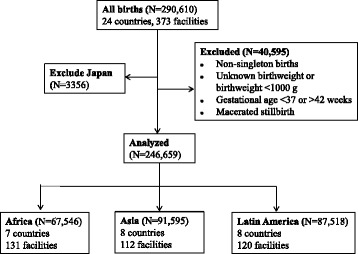
Flow chart of inclusion and exclusion of study subjects

For the purpose of this analysis, the study sample was restricted to: 1) LMICs; 2) singleton pregnancies; 3) live births or fresh stillbirth; 4) birthweight ≥ 1000 g; and 5) term births (gestational age 37–42 weeks). As the analysis focused on LMICs, Japan was excluded. Macerated stillbirths were also excluded, as we were interested in mortality associated with delivery, but not pre-delivery mortality. Infants who had missing information on birthweight or gestational age were also excluded. The sample selection process is shown in Fig. 1.

### Statistical analysis

Previous studies have shown that the perinatal mortality rate decreases with increasing birthweight or birthweight percentile until it reaches an inflection point, after which a further rise in birthweight increases the mortality, i.e., a reversed J-shaped mortality curve [13–17]. We applied this principle to our analysis: macrosomia was defined as a birthweight or birthweight percentile that exceeds the nadir of the mortality curve, and at which point the relative risk of perinatal mortality is twice or greater than that of the nadir. We used *a priori* odds ratio of 2 · 0 as a criterion for clinical significance, as per previous studies [18, 19].

We used the birthweight range of 3000–3499 g or at 50^th^–74^th^ percentiles as the reference groups because our exploratory analyses showed that the nadir of the birthweight-specific mortality rate fell into these ranges. According to the exploratory analyses, there was no significant difference of risks of neonatal mortality and morbidity among subgroups of every 100 g between 3500 and 4000 g. Thus, infants with birthweights in the range of 3500–3999 g were combined as one subgroup. Birthweights between 4000 and 4500 g were categorized into subgroups of every 100 g in order to explore the cut-off point for the definition of macrosomia.

To calculate weight percentiles, we adopted the global reference for fetal and birth weight percentile [20]. Briefly, based on the mean birthweight and variation at 40 weeks of gestation at each country, country-specific, equation-derived (i.e., not empirical) birthweight reference percentiles at 75^th^, 90^th^, 95^th^ and 97^th^ for each gestational week were generated. Infant birthweight was categorized according to these references.

The adverse maternal outcomes included maternal mortality and severe morbidity defined as any of the following: admission to an intensive-care unit (ICU), referral to a higher level or special care unit, blood transfusion, hysterectomy, vesico-vaginal/recto-vaginal fistula and third or fourth degree perineal laceration. The adverse perinatal outcomes were still-birth, early neonatal death (neonatal death within 7 days of birth) and severe neonatal morbidity defined as any of the following: admission to an Neonatal ICU for 7 days or more, referral to a higher level or special care unit and 5-min Apgar score less than four. As maternal and perinatal mortality and severe morbidity are rare outcomes, the composite maternal mortality and morbidity index (MMMI) and perinatal mortality and morbidity index (PMMI) were computed [2]. The MMMI and PMMI were coded as an event if mortality or any of the corresponding severe morbidity occurred. We also compared the risks of caesarean section and assisted vaginal delivery (forceps/vacuum extraction) for subgroups of infants by birthweight and birthweight percentile.

Two-level logistic regression models were used to estimate odds ratios (ORs) of maternal and perinatal mortality and morbidity by absolute birthweight and birthweight percentile for gestational age. Facilities represented units at level two and individuals within facilities were observations at level one. To take into account the large variations of anthropometry among regions, we stratified the analysis by region (Africa, Asia and Latin America). We adjusted for country, maternal age, marital status, education (total years of school attendance), obesity (maternal body mass index, BMI ≥30 kg/m^2^), pre-existing diabetes, parity, infant sex, and gestational age as potential confounders according to previous literature [7, 10]. All analyses were conducted with SAS version 9 · 2 (SAS Institute Inc, Cary, NC).

Maternal weight and/or height, and consequently maternal BMI, were missing in more than 10 % of the study population in Kenya (85 %), Brazil (67 %), Angola (43 %), Argentina (33 %), Uganda (21 %) and Peru (13 %). A missing category within “maternal obesity” (BMI ≥ 30 kg/m^2^) was therefore created and included in all regression models. In our sensitivity analysis, we restricted our calculations to countries with less than 10 % of the missing value for BMI. The “(pre-existing) diabetes mellitus” variable was missing in less than 1 % of the study population of each region. For the estimation of risks of MMMI and PMMI by birthweight or birthweight percentile, we performed a sensitivity analysis by excluding the variables “maternal obesity” and “diabetes” from the regression models. In another sensitivity analysis, we compared the adjusted odds ratios (aORs) of MMMI and PMMI in subgroups using birthweight percentile of the study population as cut-off points, the results were essentially unchanged (data not shown). In another sensitivity analysis, we compared the aORs of MMMI and PMMI in subgroups using the empirical country- and gestational-age-specific birthweight percentile of the study population as cut-off points, the results were essentially unchanged (not shown).

## Results

A total of 246,659 deliveries at 363 facilities in 23 LMICs were included in this analysis (Fig. 1). Large variations in birthweight distribution were observed among infants in the three regions. Mean birthweight was 3037 g, 3225 g and 3253 g in Asia, Africa and Latin America, respectively, and the proportion of infants with a birthweight greater than 4500 g was 0 · 3 %, 1 · 2 %, and 0 · 7 % across these three regions. The prevalence of birthweight greater than the 97^th^ percentile was 7.2 % (Latin America), 10.1 % (Africa) and 10 · 5 % (Asia) in the three regions (Table 1). In all three regions, the proportion of maternal age older than 35 years, maternal obesity (BMI ≥30 kg/m^2^), maternal preexisting gestational diabetes, multiparas and infant male sex was positively associated with higher birthweight (all *p* values <0 · 0001, Additional file 1: Table S1).

**Table 1:** Country-specific birthweight distribution of singleton term births

	N	Birthweight (g)			Birthweight percentile		
		Mean (SD)	≥4000 (%)	≥4500 (%)	≥P_90_ (%)	≥P_95_ (%)	≥P_97_ (%)
Africa	67546	3225 (489)	7 · 3	1 · 2	20 · 6	12 · 8	10 · 1
Angola	3304	3125 (473)	3 · 1	0 · 7	20 · 8	12 · 8	10 · 4
Algeria	14361	3469 (501)	15 · 2	3 · 2	15 · 4	9 · 1	6 · 9
Democratic Republic of Congo	6989	3077 (452)	2 · 7	0 · 3	22 · 6	15 · 4	11 · 6
Kenya	16091	3158 (448)	3 · 9	0 · 5	23 · 6	15 · 8	12 · 6
Nigeria	7721	3059 (442)	2 · 0	0 · 1	16 · 4	10 · 7	8 · 1
Niger	7296	3215 (494)	8 · 0	1 · 5	19 · 2	10 · 5	8 · 4
Uganda	11784	3252 (464)	9 · 1	0 · 8	25 · 2	14 · 6	12 · 2
Asia	91595	3037 (464)	2 · 5	0 · 3	20 · 3	13 · 8	10 · 5
Cambodia	5052	3090 (419)	2 · 5	0 · 5	16 · 6	11 · 2	9 · 1
China	13595	3333 (419)	7 · 2	0 · 7	17 · 6	10 · 0	7 · 2
India	18828	2772 (424)	0 · 6	0 · 0	28 · 2	22 · 2	16 · 6
Nepal	7316	2958 (469)	1 · 7	0 · 2	18 · 0	12 · 2	8 · 4
Philippines	11946	2961 (425)	1 · 1	0 · 2	19 · 0	12 · 2	9 · 1
Sri Lanka	13787	2980 (424)	1 · 3	0 · 1	20 · 3	13 · 9	10 · 5
Thailand	8621	3137 (437)	2 · 5	0 · 3	20 · 9	13 · 5	10 · 1
Vietnam	12450	3209 (404)	3 · 5	0 · 3	14 · 8	8 · 7	8 · 0
Latin America	87518	3253 (455)	5 · 5	0 · 7	15 · 7	9 · 7	7 · 2
Argentina	9592	3345 (453)	7 · 5	1 · 1	12 · 8	7 · 4	5 · 0
Brazil	13373	3223 (446)	4 · 4	0 · 5	14 · 8	9 · 2	6 · 7
Cuba	11817	3316 (459)	7 · 6	1 · 0	15 · 6	9 · 7	7 · 1
Ecuador	11359	3117 (440)	2 · 9	0 · 3	14 · 7	9 · 8	6 · 7
Mexico	18653	3199 (444)	4 · 1	0 · 5	17 · 5	10 · 5	8 · 2
Nicaragua	5188	3157 (435)	3 · 0	0 · 3	21 · 6	14 · 9	11 · 7
Paraguay	3051	3389 (472)	10 · 2	1 · 8	16 · 3	10 · 1	7 · 4
Peru	14485	3348 (442)	7 · 4	0 · 9	14 · 8	9 · 0	6 · 6

The overall rates of caesarean deliveries (and intrapartum rate) in Africa, Asia and Latin America were 12 · 0 % (6 · 2 %), 28 · 0 % (12 · 5 %) and 34 · 1 % (12 · 0 %) (p < 0 · 0001), respectively. Table 2 shows that compared with the reference group (3000–3499 g), aORs of elective and intrapartum caesarean section exceeded 2 · 0 when birthweight was greater than 4000 g in all three regions, while the risks of forceps or vacuum extraction did not rise significantly in most subgroups of infants in Asia and Latin America. In Africa, the aORs of forceps or vacuum extraction reached 2.0 when birthweight exceeded 4500 g. There was a large variation of elective caesarean section rate among populations. In Africa, only around 5 % suspected macrosomic babies were born through elective caesarean section while in Asia and Latin America, the responding rate was as high as around 40 and 20 %, respectively. The association between caesarean section and birthweight seemed less pronounced when infants were categorized by birthweight percentile and aORs hardly reached 2.0 (Table 3). The most important indication for CS for suspected macrosomic cases in all populations were cephalopelvic disproportion, followed by previous caesarean and fetal distress. Around 40 % of suspected macrosomic cases born through caesarean had an indication of previous caesarean section. The other two indications accounted for around 30 and 20 % caesarean deliveries, respectively (Additional file 2: Table S2).

**Table 2:** Prevalence and odds ratios of elective caesarean, intrapartum caesarean section and forceps or vacuum extraction by birthweight

Birthweight (g)		Elective caesarean section			Intrapartum caesarean section			Forceps extraction or vacuum extraction		
	N	Prevalence (%)	Adjusted OR (95 % CI)^a^	*p* value	Prevalence (%)	Adjusted OR (95 % CI)^a^	*p* value	Prevalence (%)	Adjusted OR (95 % CI)^a^	*p* value
Africa	67546									
3000–3499	28788	1.9	1 · 00	–	5 · 8	1 · 00	–	1 · 8	1 · 00	–
3500–3999	15824	2.3	1.19 (1.03, 1.39)	0.0196	6 · 4	1 · 27 (1 · 16, 1 · 38)	<0 · 0001	2 · 4	1 · 22 (1 · 05, 1 · 41)	0 · 0094
4000–4099	2154	2.1	1.14 (0.79, 1.63)	0.4824	9 · 0	2 · 00 (1 · 68, 2 · 39)	<0 · 0001	2 · 9	1 · 81 (1 · 35, 2 · 44)	0 · 0001
4100–4199	670	4.2	1.96 (1.25, 3.07)	0.0032	8 · 1	1 · 87 (1 · 38, 2 · 53)	0 · 0001	3 · 4	1 · 64 (1 · 03, 2 · 6)	0 · 0358
4200–4299	686	5.0	2.61 (1.72, 3.96)	<0 · 0001	9 · 6	2 · 36 (1 · 78, 3 · 12)	<0 · 0001	2 · 2	1 · 09 (0 · 63, 1 · 88)	0 · 7618
4300–4399	377	2.0	0.83 (0.36, 1.93)	0.6687	10 · 6	2 · 39 (1 · 67, 3 · 42)	<0 · 0001	2 · 7	0 · 98 (0 · 50, 1 · 92)	0 · 9555
4400–4499	239	5.7	3.26 (1.67, 6.34)	0.0005	8 · 8	2 · 48 (1 · 52, 4 · 04)	0 · 0003	3 · 3	1 · 84 (0 · 86, 3 · 93)	0 · 1131
4500–4999	648	6.3	3.62 (2.40, 5.46)	<0 · 0001	13 · 0	3 · 78 (2 · 90, 4 · 92)	<0 · 0001	3 · 7	2 · 17 (1 · 37, 3 · 42)	0 · 0009
≥ 5000	146	5.0	2.99 (1.12, 7.95)	0.0287	14 · 4	5 · 11 (3 · 00, 8 · 72)	<0 · 0001	8 · 9	8 · 34 (4 · 22, 16 · 5)	<0 · 0001
Asia	91595									
3000–3499	36479	11.8	1 · 00	–	13 · 2	1 · 00	–	2 · 7	1 · 00	–
3500–3999	13539	18.3	1.36 (1.27, 1.47)	<0 · 0001	18 · 6	1 · 44 (1 · 36, 1 · 53)	<0 · 0001	2 · 6	1 · 27 (1 · 12, 1 · 45)	0 · 0003
4000–4099	945	31.6	2.33 (1.90, 2.86)	<0 · 0001	24 · 1	2 · 42 (2 · 02, 2 · 89)	<0 · 0001	2 · 1	1 · 64 (1 · 02, 2 · 64)	0 · 0413
4100–4199	492	34.8	2.32 (1.76, 3.07)	<0 · 0001	26 · 6	2 · 61 (2 · 05, 3 · 33)	<0 · 0001	2 · 2	1 · 69 (0 · 87, 3 · 29)	0 · 1195
4200–4299	320	40.3	3.19 (2.28, 4.47)	<0 · 0001	28 · 1	3 · 79 (2 · 77, 5 · 18)	<0 · 0001	3 · 1	2 · 73 (1 · 37, 5 · 46)	0 · 0045
4300–4399	183	40.5	2.63 (1.70, 4.07)	<0 · 0001	30 · 1	3 · 29 (2 · 22, 4 · 88)	<0 · 0001	2 · 2	2 · 56 (0 · 87, 7 · 55)	0 · 0884
4400–4499	101	47.6	3.45 (1.90, 6.25)	<0 · 0001	29 · 7	4 · 13 (2 · 38, 7 · 15)	<0 · 0001	2 · 0	1 · 92 (0 · 42, 8 · 67)	0 · 3969
4500–4999	200	50.0	5.48 (3.45, 8.70)	<0 · 0001	30 · 0	5 · 20 (3 · 45, 7 · 83)	<0 · 0001	2 · 0	2 · 47 (0 · 81, 7 · 57)	0 · 1124
≥ 5000	52	40.0	4.20 (1.71, 10.34)	0.0018	32 · 7	9 · 44 (3 · 99, 22 · 35)	<0 · 0001	1 · 9	2 · 38 (0 · 3, 18 · 81)	0 · 4113
Latin America	87518									
3000–3499	38930	15.3	1 · 00	–	11 · 2	1 · 00	–	1 · 2	1 · 00	–
3500–3999	20875	16.9	1.19 (1.13, 1.26)	<0 · 0001	14 · 0	1 · 28 (1 · 21, 1 · 35)	<0 · 0001	1 · 5	1 · 51 (1 · 30, 1 · 77)	<0 · 0001
4000–4099	1675	21.7	1.64 (1.40, 1.91)	<0 · 0001	18 · 1	2 · 01 (1 · 74, 2 · 33)	<0 · 0001	0 · 7	0 · 80 (0 · 43, 1 · 48)	0 · 4699
4100–4199	1020	23.0	1.92 (1.58, 2.34)	<0 · 0001	19 · 1	2 · 19 (1 · 83, 2 · 62)	<0 · 0001	1 · 3	1 · 45 (0 · 79, 2 · 65)	0 · 2318
4200–4299	757	25.1	2.01 (1.60, 2.51)	<0 · 0001	21 · 7	2 · 48 (2 · 03, 3 · 03)	<0 · 0001	1 · 5	1 · 88 (0 · 99, 3 · 58)	0 · 0551
4300–4399	466	22.6	1.95 (1.45, 2.61)	<0 · 0001	24 · 4	3 · 00 (2 · 35, 3 · 84)	<0 · 0001	0 · 9	0 · 90 (0 · 32, 2 · 50)	0 · 8354
4400–4499	281	28.7	2.59 (1.83, 3.66)	<0 · 0001	19 · 6	2 · 66 (1 · 89, 3 · 73)	<0 · 0001	1 · 1	1 · 81 (0 · 55, 5 · 94)	0 · 3257
4500–4999	569	38.1	3.66 (2.87, 4.68)	<0 · 0001	23 · 8	4 · 17 (3 · 29, 5 · 3)	<0 · 0001	1 · 1	1 · 98 (0 · 84, 4 · 63)	0 · 1163
≥ 5000	55	35.3	3.54 (1.67, 7.53)	0.0010	20 · 0	3 · 13 (1 · 43, 6 · 85)	0 · 0045	3 · 6	4 · 64 (0 · 95, 22 · 64)	0 · 0579

^a^All estimates were based on two-level logistic regression models. Facilities represent units at level two and individuals within facilities are observations at level one. We adjusted for country, maternal age, marital status, education (total years of school attendance), obesity, diabetes, parity, infant sex, and gestational age

**Table 3:** Prevalence and odds ratios of elective caesarean, intrapartum caesarean section and forceps or vacuum extraction by birthweight percentile

Birthweight percentile		Elective caesarean section			Intrapartum caesarean section			Forceps extraction or vacuum extraction		
	N	Prevalence (%)	Adjusted OR (95 % CI)^a^	*p* value	Prevalence (%)	Adjusted OR (95 % CI)^a,^	*p* value	Prevalence (%)	Adjusted OR (95 % CI)^a,^	*p* value
Africa	67546									
P_50−_P_74_	15747	1.8	1 · 00	–	5 · 5	1 · 00	–	1 · 9	1 · 00	–
P_75_–P_89_	10150	2.3	1.31 (1.08, 1.59)	0.0067	6 · 5	1 · 23 (1 · 10, 1 · 38)	0 · 0002	1 · 8	1 · 18 (0 · 97, 1 · 44)	0 · 1032
P_90_–P_94_	5247	2.5	1.36 (1.08, 1.72)	0.0093	6 · 0	1 · 25 (1 · 09, 1 · 44)	0 · 0019	1 · 8	1 · 39 (1 · 08, 1 · 78)	0 · 0106
P_95_–P_96_	1820	3.6	1.91 (1.40, 2.60)	<0 · 0001	6 · 8	1 · 46 (1 · 18, 1 · 79)	0 · 0004	1 · 2	1 · 02 (0 · 64, 1 · 62)	0 · 9335
≥ P_97_	6851	3.8	2.00 (1.64, 2.44)	<0 · 0001	9 · 0	2 · 17 (1 · 92, 2 · 44)	<0 · 0001	1 · 7	1 · 86 (1 · 46, 2 · 37)	<0 · 0001
Asia	91595									
P_50_–P_74_	18066	12.3	1 · 00	–	12 · 5	1 · 00	–	2 · 6	1 · 00	–
P_75_–P_89_	14694	12.1	1.08 (1.00, 1.18)	0.0611	12 · 8	1 · 11 (1 · 03, 1 · 19)	0 · 0056	2 · 5	1 · 08 (0 · 93, 1 · 25)	0 · 3198
P_90_–P_94_	5955	16.1	1.22 (1.10, 1.36)	0.0002	14 · 9	1 · 37 (1 · 25, 1 · 51)	<0 · 0001	2 · 2	1 · 05 (0 · 86, 1 · 29)	0 · 6286
P_95_–P_96_	3030	14.5	1.31 (1.14, 1.51)	0.0002	13 · 4	1 · 60 (1 · 41, 1 · 82)	<0 · 0001	3 · 2	1 · 67 (1 · 32, 2 · 11)	<0 · 0001
≥ P_97_	9574	18.1	1.65 (1.50, 1.81)	<0 · 0001	17 · 2	1 · 90 (1 · 75, 2 · 06)	<0 · 0001	3 · 0	1 · 55 (1 · 31, 1 · 83)	<0 · 0001
Latin America	87518									
P_50_–P_74_	20003	15.8	1 · 00	–	12 · 3	1 · 00	–	1 · 3	1 · 00	–
P_75_–P_89_	12912	17.7	1.11 (1.03, 1.19)	0.0062	12 · 7	1 · 08 (1 · 01, 1 · 17)	0 · 0291	1 · 3	1 · 13 (0 · 91, 1 · 39)	0 · 2628
P_90_–P_94_	5227	19.9	1.27 (1.16, 1.40)	<0 · 0001	13 · 8	1 · 33 (1 · 21, 1 · 47)	<0 · 0001	1 · 7	1 · 55 (1 · 20, 2 · 02)	0 · 0010
P_95_–P_96_	2242	21.7	1.42 (1.24, 1.63)	<0 · 0001	15 · 0	1 · 45 (1 · 26, 1 · 66)	<0 · 0001	1 · 2	1 · 34 (0 · 88, 2 · 06)	0 · 1770
≥ P_97_	6284	25.2	1.65 (1.51, 1.80)	<0 · 0001	15 · 5	1 · 84 (1 · 68, 2 · 02)	<0 · 0001	1 · 2	1 · 34 (1 · 01, 1 · 78)	0 · 0402

^a^All estimates were based on two-level logistic regression models. Facilities represent units at level two and individuals within facilities are observations at level one. We adjusted for country, maternal age, marital status, education (total years of school attendance), obesity, diabetes, parity, infant sex and gestational age

**Fig. 2:**
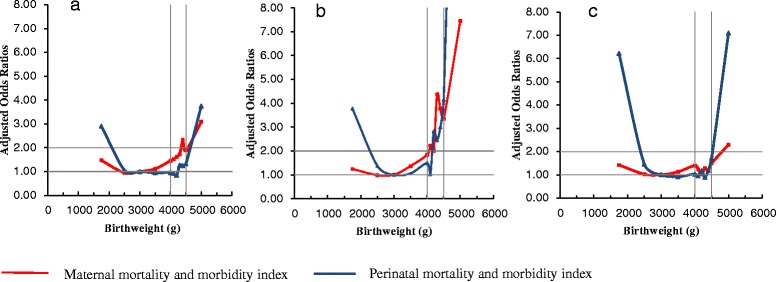
Adjusted odds ratios of maternal and perinatal mortality and morbidity by birthweight in singleton term births. **a**: Africa **b**: Asia **c**: Latin America reference category: 3000 –3499 g

The birthweight-specific risks of MMMI and PMMI are presented in Fig. 2. The association is expressed in a reverse “J”- or “U”-shaped curves. In all three regions, the lowest risk of MMMI corresponded to the birthweight range of 3000–3500 g, which was used as the reference group. When the birthweight exceeded 3500 g, aORs of MMMI increased gradually. The aORs of MMMI reached 2 · 0 when birthweight was greater than 4000 g, 4500 g and 5000 g in Asia, Africa and Latin America, respectively. The rise in PMMI risks lagged behind that of MMMI in all three regions (Fig. 2). The aORs of PMMI reached 2 · 0 when birthweight was greater than 4200 g in Asia and 5000 g in Africa. In Latin America, birthweight of 4500–4999 g corresponded to the aOR of PMMI 1 · 78 (95 % CI: 1 · 16, 2 · 74). When birthweight was greater than 5000 g, aOR rose dramatically to 7 · 40 (95 % CI: 3 · 5, 15 · 66) (Table 4).

**Table 4:** Prevalence and odds ratios of maternal and perinatal mortality and morbidity by birthweight

Birthweight (g)	N	Maternal mortality and morbidity			Perinatal mortality and morbidity		
		Prevalence (%)	Adjusted OR (95 % CI)^a^	*p* value	Prevalence (%)	Adjusted OR (95 % CI)^a^	*p* value
Africa	67546		N = 67546				
3000–3499	28788	6 · 0	1 · 00	–	4 · 6	1 · 00	–
3500–3999	15824	7 · 2	1 · 10 (1 · 01, 1 · 20)	0 · 0289	4 · 2	0 · 94 (0 · 85, 1 · 05)	0 · 2718
4000–4099	2154	9 · 1	1 · 43 1 · 20, 1 · 70)	0 · 0001	4 · 0	0 · 97 (0 · 76, 1 · 23)	0 · 7780
4100–4199	670	10 · 6	1 · 48 (1 · 12, 1 · 96)	0 · 0061	3 · 4	0 · 93 (0 · 60, 1 · 44)	0 · 7424
4200–4299	686	11 · 7	1 · 59 (1 · 22, 2 · 07)	0 · 0006	3 · 4	0 · 84 (0 · 55, 1 · 31)	0 · 4473
4300–4399	377	14 · 3	1 · 68 (1 · 21, 2 · 34)	0 · 0021	5 · 0	1 · 29 (0 · 79, 2 · 09)	0 · 3122
4400–4499	239	15 · 5	2 · 26 (1 · 51, 3 · 39)	0 · 0001	3 · 8	1 · 26 (0 · 63, 2 · 49)	0 · 5147
4500–4999	648	15 · 0	1 · 84 (1 · 44, 2 · 36)	<0 · 0001	5 · 4	1 · 34 (0 · 93, 1 · 93)	0 · 1216
≥ 5000	146	21 · 2	3 · 01 (1 · 91, 4 · 74)	<0 · 0001	14 · 4	3 · 79 (2 · 28, 6 · 30)	<0 · 0001
Asia	91595		N = 91595				
3000–3499	36479	4 · 7	1 · 00	–	1 · 7	1 · 00	–
3500–3999	13539	5 · 4	1 · 38 (1 · 24, 1 · 52)	<0 · 0001	1 · 6	1 · 05 (0 · 90, 1 · 24)	0 · 5196
4000–4099	945	5 · 4	1 · 86 (1 · 35, 2 · 56)	0 · 0002	2 · 1	1 · 49 (0 · 94, 2 · 35)	0 · 0891
4100–4199	492	6 · 7	2 · 25 (1 · 50, 3 · 38)	0 · 0001	1 · 2	1 · 01 (0 · 45, 2 · 29)	0 · 9812
4200–4299	320	5 · 0	2 · 03 (1 · 17, 3 · 52)	0 · 0122	4 · 1	2 · 80 (1 · 58, 4 · 99)	0 · 0005
4300–4399	183	11 · 5	4 · 42 (2 · 60, 7 · 50)	<0 · 0001	3 · 3	2 · 44 (1 · 06, 5 · 64)	0 · 0366
4400–4499	101	7 · 9	3 · 81 (1 · 68, 8 · 60)	0 · 0013	4 · 0	2 · 99 (1 · 07, 8 · 34)	0 · 0367
4500–4999	200	10 · 0	3 · 38 (1 · 99, 5 · 74)	<0 · 0001	6 · 0	4 · 12 (2 · 24, 7 · 56)	<0 · 0001
≥ 5000	52	17 · 3	7 · 51 (3 · 25, 17 · 33)	<0 · 0001	26 · 9	28 · 44 (14 · 81, 54 · 61)	<0 · 0001
Latin America	87518		N = 87518				
3000–3499	38930	2 · 4	1 · 00	–	2 · 1	1 · 00	–
3500–3999	20875	2 · 6	1 · 15 (1 · 03, 1 · 28)	0 · 0157	2 · 0	0 · 94 (0 · 83, 1 · 06)	0 · 3390
4000–4099	1675	3 · 0	1 · 44 (1 · 08, 1 · 94)	0 · 0145	2 · 3	1 · 07 (0 · 77, 1 · 49)	0 · 6852
4100–4199	1020	2 · 6	1 · 28 (0 · 86, 1 · 90)	0 · 2229	2 · 2	0 · 98 (0 · 64, 1 · 52)	0 · 9422
4200–4299	757	2 · 5	1 · 05 (0 · 65, 1 · 70)	0 · 8450	2 · 8	1 · 22 (0 · 78, 1 · 91)	0 · 3719
4300–4399	466	3 · 0	1 · 34 (0 · 77, 2 · 31)	0 · 3014	2 · 1	0 · 94 (0 · 5, 1 · 77)	0 · 8429
4400–4499	281	2 · 8	1 · 19 (0 · 58, 2 · 46)	0 · 6381	2 · 8	1 · 25 (0 · 61,2 · 56)	0 · 5378
4500–4999	569	3 · 5	1 · 54 (0 · 96, 2 · 45)	0 · 0708	4 · 0	1 · 78 (1 · 16, 2 · 74)	0 · 0089
≥ 5000	55	5 · 5	2 · 36 (0 · 70, 8 · 00)	0 · 1671	16 · 4	7 · 40 (3 · 50, 15 · 66)	<0 · 0001

^a^All estimates were based on two-level logistic regression models. Facilities represent units at level two and individuals within facilities are observations at level one. We adjusted for country, maternal age, marital status, education (total years of school attendance), obesity, diabetes, parity, infant sex, and gestational age

When we excluded prelabor caesarean deliveries or restricted the analyses to vaginal deliveries, the results remained essentially unchanged (Additional file 3: Tables S3 and S4).

Table 5 shows that the risks of MMMI and PMMI in infants with a birthweight greater than the 95^th^ percentile increased slightly compared with that of birthweight at the 50^th^–75^th^ percentiles in all three regions. When birthweight was at the 97^th^ percentile or higher, the aORs of MMMI and PMMI increased significantly, but none exceeded 2 · 0 in any region. In addition to the equation-derived global reference birthweight percentiles, we also used the empirical country- and gestational-age-specific birthweight percentile of the study population as cut-off points. The results were essentially unchanged (not shown).

**Table 5:** Prevalence and odds ratios of maternal and perinatal mortality and morbidity by birthweight percentile

Birthweight percentile	N	Maternal mortality and morbidity			Perinatal mortality and morbidity		
		Prevalence (%)	Adjusted OR (95 % CI)^a^	*p* value	Prevalence (%)	Adjusted OR (95 % CI)^a,^	*p* value
Africa	67546						
P_50_–P_74_	15747	6 · 3	1 · 00	–	4 · 3	1 · 00	–
P_75_–P_89_	10150	6 · 6	1 · 17 (1 · 05, 1 · 31)	0 · 0056	4 · 3	0 · 98 (0 · 86, 1 · 11)	0 · 7055
P_90_–P_94_	5247	5 · 7	1 · 07 (0 · 93, 1 · 24)	0 · 3383	5 · 0	1 · 07 (0 · 92, 1 · 25)	0 · 3900
P_95_–P_96_	1820	6 · 6	1 · 31 (1 · 05, 1 · 62)	0 · 0144	5 · 4	1 · 15 (0 · 91, 1 · 45)	0 · 2301
≥ P_97_	6851	7 · 2	1 · 54 (1 · 36, 1 · 75)	<0 · 0001	5 · 8	1 · 10 (0 · 96, 1 · 26)	0 · 1794
Asia	91595						
P_50_–P_74_	18066	5 · 0	1 · 00	–	1 · 8	1 · 00	–
P_75_–P_89_	14694	5 · 1	1 · 03 (0 · 92, 1 · 15)	0 · 5834	2 · 0	1 · 02 (0 · 86, 1 · 19)	0 · 8529
P_90_–P_94_	5955	4 · 8	1 · 04 (0 · 89, 1 · 21)	0 · 6389	1 · 7	0 · 88 (0 · 70, 1 · 11)	0 · 2784
P_95_–P_96_	3030	5 · 1	1 · 20 (0 · 98, 1 · 46)	0 · 0742	2 · 4	1 · 08 (0 · 83, 1 · 41)	0 · 5513
≥ P_97_	9574	6 · 4	1 · 41 (1 · 25, 1 · 59)	<0 · 0001	2 · 5	1 · 12 (0 · 94, 1 · 34)	0 · 1909
Latin America	87518						
P_50_–P_74_	20003	2 · 4	1 · 00	–	2 · 0	1 · 00	–
P_75_–P_89_	12912	2 · 7	1 · 13 (0 · 98, 1 · 30)	0 · 1014	2 · 2	1 · 10 (0 · 94, 1 · 29)	0 · 2236
P_90_–P_94_	5227	2 · 6	1 · 06 (0 · 87, 1 · 29)	0 · 5709	1 · 8	0 · 89 (0 · 71, 1 · 12)	0 · 3257
P_95_–P_96_	2242	2 · 9	1 · 21 (0 · 92, 1 · 58)	0 · 1744	2 · 1	0 · 98 (0 · 72, 1 · 34)	0 · 9162
≥ P_97_	6284	3 · 0	1 · 23 (1 · 03, 1 · 47)	0 · 0215	2 · 8	1 · 31 (1 · 09, 1 · 58)	0 · 0042

^a^All estimates based on two-level logistic regression models. Facilities represent units at level two and individuals within facilities are observations at level one. We adjusted for country, maternal age, marital status, education (total years of school attendance), obesity, diabetes, parity, infant sex, and gestational age

We conducted sensitivity analyses using mortality and morbidity as two separate outcomes. The mortality included maternal and perinatal deaths. For morbidity, the occurrence of any components of MMMI and PMMI (excluding maternal or perinatal death) was considered as a positive event. As maternal and perinatal mortality were rare, no significant differences in mortality were found in the subgroups of infants with a birthweight greater than 4500 g, or the 97^th^ birthweight percentile compared with the reference groups. However, results were similar for maternal and perinatal morbidity after excluding maternal or perinatal death (data no shown). Similar trends for the risks of MMMI and PMMI by birthweight and birthweight percentile were found when we restricted the analyses to countries with less than 10 % of missing value for BMI (Additional file 3: Tables S5 and S6). When maternal obesity and diabetes were excluded from the regression models, aORs became larger for most of the subgroups, but the patterns remained the same in the three regions (Additional file 3: Tables S7 and S8).

## Discussion

Our results indicate that there is a significant increase in adverse maternal and perinatal outcomes when the birthweight of term infants (37–42 weeks) reaches 4500 g in African and Latin America, and 4000 g in Asia. These cut-offs could, therefore, be used to define ‘macrosomia’ in these settings. Our findings do not support using LGA as a new definition for macrosomia because LGA was less predictive of adverse outcomes.

The use of the cut-off point of 4500 g to define macrosomia is consistent with results of previous studies of Caucasians populations [1, 9], and supports the definition of the American Congress of Obstetricians and Gynecologists [1]. Based on analyses of national datasets of the United States, Zhang et al. [7], found that infants with birthweight of 4500–4999 g were at significantly increased risks of stillbirth, neonatal mortality (especially because of birth asphyxia), morbidity, and caesarean delivery as a consequence of either slow labor progress or non-reassuring cardiotocography. Ye et al. [9] used the same database also found that risks of MMMI or PMMI did not increased significantly until birthweight was at the 97^th^ percentile or higher. A birthweight cut-off points irrespective of gestational age (4500 g in Whites, 4300 g in Blacks and Hispanics) is more predictive of mortality and morbidity outcomes than the 97^th^ percentile for a given gestational age.

We used an OR of 2 · 0 for either MMMI or PMMI as *a priori* criterion to identify clinically important macrosomia. Though arbitrary, this cut-off point was also used in a study by Boulet et al. [18] in defining clinically important fetal growth restriction. In the randomized trial of the Twin Birth Study Collaborative Group, a relative risk of 0 · 5 was also used to justify the smallest clinically important difference between the planned caesarean delivery group and control group [19], equivalent to two-fold increase (or decrease) of risks.

Using the concept of birthweight percentile at a given gestational age (i.e., LGA) as the definition of macrosomia has been proposed in recent years [2, 10, 21]. However, our study shows that it has a poor prediction of adverse maternal and perinatal outcomes. This may be partially attributable to the imprecise estimation of gestational age, which has resulted in misclassifications of macrosomia. Thus, a definition based on birthweight would be more practical, especially in settings where accurate estimation of gestational age may be difficult.

We observed that risks of elective and intrapartum caesarean section increased significantly for infants with a birthweight greater than 4000 g in the selected facilities of all three regions. However, the risks of or vacuum extraction did not increase prominently, which suggested that obstetricians may have a tendency for operative delivery when faced with a suspiciously large fetus and slow labor progress. Therefore, an estimated birthweight of 4000 g may be a useful indicator for difficult labour. We also found that risks of MMMI and PMMI did not change substantially after excluding elective caesarean deliveries, suggesting that suspected macrosomic cases may not benefits from elective caesarean section in a meaningful way. This was also demonstrated in other studies [22, 23].

We also observed that risks of MMMI increased prior to that of PMMI in three regions. This was consistent with the three-level definition of macrosomia proposed by Boulet et al. [3]: Grade 1 (>4000 g) to identify increased risks of labour and newborn complications; Grade 2 (>4500 g) to predict neonatal morbidity; and Grade 3 (>5000 g) to predict infant mortality. The major causes of maternal mortality and morbidity for mothers of macrosomic babies included uterine atony, prolonged labour, haemorrhage, vesico-vaginal/recto-vaginal fistula and severe perineal laceration [21]. These complications contributed to MMMI in our study. The most frequently severe adverse outcome was admission to an intensive-care unit (5.0 %), followed by blood transfusion (2.0 %) and third or fourth degree perineal laceration (1.5 %) for suspected macrosomia infants.

Macrosomia represents a significant obstetric challenge. The definition of macrosomia has important clinical, medicolegal and cost implications. Therefore, it should be evidence-based, particularly in resources limited countries where skilled birth attendants and caesarean delivery may not be readily available, and hospital transfer and special care are costly. To the best of our knowledge, this is the first study to search for an evidence-based definition of macrosomia in LMICs.

However, our study has several limitations. First, the significance of the definition of macrosomia using birthweight cut-off point in obstetric management is limited by the inaccuracy in birthweight estimation. Either ultrasound or clinical prediction of birthweight is not accurate enough to serve as the basis for obstetric decision making. But obstetricians are prone to operative delivery when faced with a suspected large fetus, which may lead to unnecessary cesarean [24].

Second, despite the standardization of data collection, participating facilities may have different labour management protocols. Inter-institutional variability is inevitable due to the nature of a multinational study. For example, screening for diabetes in pregnancy is not available in all facilities, particularly in Africa [2], and therefore underestimation of gestational diabetes is likely. However, our sensitivity analysis, which excluded the variable “diabetes” from the regression models, showed a similar result to that of the fully adjusted models.

Third, information on maternal height and weight was problematic in two aspects. More than 10 % of data for height and/or weight were missing for some countries. In addition, maternal weight was defined differently across regions: in Africa and Latin America it was referred to as the first recorded weight at the first antenatal care visit while in Asia it was defined as the last recorded weight before delivery. In theory, the last recorded maternal weight would better control for the potential confounding effects of gestational weight gain in Asia [25]. However, the results of the sensitivity analysis, which excluded those countries or the variable “obesity” from the regression model indicated that the limitation did not affect the conclusion.

Finally, the sample used in the survey was selected from facilities with more than 1000 deliveries per year and where caesarean sections were available [11]. Selection bias is possible, especially in certain LMICs where an institutional delivery rate is low. Therefore, the generalizability of our findings may be limited.

## Conclusions

A population-specific definition of macrosomia using birthweight cut-off points (4500 g in Africa and Latin America, 4000 g in Asia) for term infants at 37–42 gestational weeks is more closely associated with maternal and perinatal mortality and morbidity. This definition is also easier to apply than that based on birthweight percentile for a given gestational age. The use of an evidence-based definition of macrosomia may improve obstetric and perinatal care, especially in resource-limited settings in LMICs.

## Additional files

Additional file 1: Table S1. Maternal characteristics by region and birthweight in singleton term births. (PDF 365 kb) Additional file 2: Table S2. Indications for caesarean section in suspected macrosomic infants. (PDF 187 kb) Additional file 3: Tables S3–S8. Results of sensitivity analyses. (PDF 577 kb)

## Abbreviations

aORs: adjusted odds ratios
BMI: body mass index
ICU: intensive-care unit
LGA: large-for-gestational-age
LMICs: low- and middle-income countries
MMMI: maternal mortality and morbidity index
ORs: odds ratios
PMMI: perinatal mortality and morbidity index
