# Supplementary material for: Searching for the definition of macrosomia through an outcome-based approach in low- and middle-income countries: a secondary analysis of the WHO Global Survey in Africa, Asia and Latin America
Source: BMC Pregnancy Childbirth. 2015 Dec 3;15:324. doi: 10.1186/s12884-015-0765-z (PMC4669645; doi:10.1186/s12884-015-0765-z)
Supplement: Additional file 1: Table S1. — Maternal characteristics by region and birthweight in singleton term births. (PDF 365 kb) [file 12884_2015_765_MOESM1_ESM.pdf]

Additional Table 1 Maternal characteristics by region and birthweight in singleton term births

Birthweight	n	Age (≥ 35 years, %)	Marital status (married/cohabiting, %)	Education (≥ 13 years, %)	Obesity (%)[a]	Diabetes (%)[b]	Parity (>1, %)	Infant sex (male, %)
**Africa**	**67546**	**13·6**	**88·6**	**13·1**	**13·2**	**0·4**	**68·2**	**51·4**
<2500	3267	10·4	83·4	8·2	6·3	0·4	54·3	43·1
2500–2999	14747	10·5	84·5	10·9	7·5	0·3	59·8	44·8
3000–3499	28788	12·4	88·5	12·9	11·5	0·3	67·9	51·0
3500–3999	15824	16·3	91·6	15·5	18·7	0·4	74·7	57·0
4000–4499	4126	21·2	94·9	16·5	25·8	1·1	82·0	60·2
4500–4999	648	28·4	95·4	17·4	35·5	1·5	85·2	63·0
≥5000	146	43·2	99·3	11·6	43·8	5·5	92·4	66·4
**Asia**	**91595**	**8·7**	**93·5**	**17·5**	**9·8**	**0·8**	**56·8**	**52·2**
<2500	7723	7·6	91·2	11·4	5·2	0·8	49·3	45·0
2500–2999	31561	7·9	91·9	14·4	6·5	0·7	52·5	47·4
3000–3499	36479	8·8	94·0	19·4	10·1	0·7	58·7	53·9
3500–3999	13539	10·0	96·4	22·4	16·1	1·2	64·0	60·4
4000–4499	2041	12·4	97·4	23·4	26·9	2·6	72·6	66·4
4500–4999	200	18·0	96·5	17·5	42·0	4·5	74·5	67·5
≥5000	52	28·9	92·3	26·9	30·8	9·6	57·7	63·5
**Latin America**	**87518**	**10·4**	**77·2**	**10·8**	**22·2**	**0·7**	**65·5**	**50·2**
<2500	3516	9·6	74·0	9·8	15·2	0·5	58·9	43·0
2500–2999	19374	9·0	74·7	9·7	16·3	0·6	60·3	42·7
3000–3499	38930	9·9	77·2	10·7	20·7	0·6	64·8	49·4
3500–3999	20875	11·4	79·5	11·6	27·7	0·8	70·2	56·8
4000–4499	4199	14·8	80·2	12·6	37·1	1·7	76·1	63·1
4500–4999	569	19·2	79·4	13·7	48·7	4·1	83·8	64·0
≥5000	55	30·9	81·8	18·2	43·6	9·1	81·8	67·3

All variables differed significantly by birthweight categories (χ^2_{CMH}, p <0·0001)·

a: Obesity is defined as body mass index ≥30 kg/m^2.

b: Pre-existing diabetes.
