# Supplementary material for: Searching for the definition of macrosomia through an outcome-based approach in low- and middle-income countries: a secondary analysis of the WHO Global Survey in Africa, Asia and Latin America
Source: BMC Pregnancy Childbirth. 2015 Dec 3;15:324. doi: 10.1186/s12884-015-0765-z (PMC4669645; doi:10.1186/s12884-015-0765-z)
Supplement: Additional file 2: Table S2. — Indications for caesarean section in suspected macrosomic infants. (PDF 187 kb) [file 12884_2015_765_MOESM2_ESM.pdf]

Additional Table 2 Top three indications for caesarean section in suspected macrosomic infants

	Africa (%)	Asia (%)	Latin America (%)
**Birthweight (g)**			
4000–4499	Cephalopelvic disproportion (47.9)	Cephalopelvic disproportion (41.2)	Cephalopelvic disproportion (44.2)
	Fetal distress (27.9)	Any other fetal indication (18.8)	Previous caesarean section (27.5)
	Previous caesarean section (26.5)	Maternal request (16.9)	Fetal distress (18.6)
4500–4999	Cephalopelvic disproportion (51.5)	Cephalopelvic disproportion (47.2)	Cephalopelvic disproportion (48.5)
	Previous caesarean section (27.5)	Any other fetal indication (31.7)	Previous caesarean section (27.0)
	Fetal distress (25.4)	Fetal distress (15.5)	Any other fetal indication (18.7)
≥5000	Cephalopelvic disproportion (59.2)	Cephalopelvic disproportion (52.9)	Cephalopelvic disproportion (72.7)
	Previous caesarean section (36.7)	Any other fetal indication (26.5)	Any other fetal indication (30.3)
	Any other fetal indication (26.5)	Maternal request (17.7)	Previous caesarean section (21.2)
**Birthweight percentile**			
P90–P94	Cephalopelvic disproportion (41.7)	Cephalopelvic disproportion （29.0）	Previous caesarean section （34.6）
	Fetal distress (27.1)	Previous caesarean section （25.9）	Cephalopelvic disproportion （31.3）
	Previous caesarean section (26.3)	Fetal distress （16.4）	Fetal distress （17.3）
P95–P96	Cephalopelvic disproportion (36.9)	Cephalopelvic disproportion （28.0）	Cephalopelvic disproportion （35.4）
	Fetal distress (26.6)	Previous caesarean section （26.7）	Previous caesarean section （34.0）
	Previous caesarean section (26.2)	Fetal distress （18.6）	Fetal distress （18.2）
≥P97	Cephalopelvic disproportion (49.0)	Cephalopelvic disproportion （32.8）	Cephalopelvic disproportion （37.5）
	Previous caesarean section (27.3)	Previous caesarean section （28.4）	Previous caesarean section （33.2）
	Fetal distress (24.1)	Fetal distress （16.3）	Fetal distress （15.7）
