# Supplementary material for: Searching for the definition of macrosomia through an outcome-based approach in low- and middle-income countries: a secondary analysis of the WHO Global Survey in Africa, Asia and Latin America
Source: BMC Pregnancy Childbirth. 2015 Dec 3;15:324. doi: 10.1186/s12884-015-0765-z (PMC4669645; doi:10.1186/s12884-015-0765-z)
Supplement: Additional file 3: Tables S3–S8. — Results of sensitivity analyses. (PDF 577 kb) [file 12884_2015_765_MOESM3_ESM.pdf]

Additional Table 3 Prevalence and adjusted odds ratios of maternal and perinatal mortality and morbidity by birthweight in singleton term infants excluding pre-labor cesarean deliveries

	Maternal mortality and morbidity		Perinatal mortality and morbidity	
**Birthweight (g)**	**Prevalence (%)**	**Adjusted OR (95% CI)[a]**	**Prevalence (%)**	**Adjusted OR (95% CI)[a]**
**Africa**	**N=63325**			
3000–3499	4·4	1·00	4·3	1·00
3500–3999	5·4	1·14 (1·02, 1·26)	3·9	0·93 (0·83, 1·04)
4000–4099	7·5	1·53 (1·25, 1·88)	3·7	0·97 (0·75, 1·26)
4100–4199	7·3	1·41 (0·99, 2·01)	3·3	0·96 (0·60, 1·53)
4200–4299	7·5	1·46 (1·04, 2·04)	2·9	0·80 (0·49, 1·30)
4300–4399	9·1	1·45 (0·95, 2·22)	4·4	1·22 (0·71, 2·10)
4400–4499	10·9	2·07 (1·24, 3·47)	3·5	1·29 (0·60, 2·80)
4500–4999	9·8	1·50 (1·08, 2·07)	5·0	1·35 (0·90, 2·03)
≥5000	16·2	3·20 (1·82, 5·60)	17·1	5·12 (3·01, 8·72)
**Asia**	**N=77399**			
3000–3499	4·7	1·00	1·6	1·00
3500–3999	5·6	1·38 (1·24, 1·55)	1·4	1·05 (0·87, 1·26)
4000–4099	6·2	2·04 (1·42, 2·92)	1·9	1·42 (0·81, 2·50)
4100–4199	8·0	2·32 (1·47, 3·68)	1·2	1·03 (0·38, 2·81)
4200–4299	5·6	2·46 (1·29, 4·69)	3·3	2·49 (1·15, 5·38)
4300–4399	12·9	4·80 (2·61, 8·81)	4·8	3·61 (1·54, 8·46)
4400–4499	9·5	4·32 (1·65, 11·35)	6·3	5·24 (1·83, 14·97)
4500–4999	11·9	3·47 (1·81, 6·62)	7·6	5·38 (2·64, 10·95)
≥5000	22·9	7·97 (3·07, 20·71)	28·6	32·11 (14·53, 70·96)
**Latin America**	**N=67782**			
3000–3499	2·2	1·00	1·9	1·00
3500–3999	2·3	1·12 (0·98, 1·28)	2·0	0·96 (0·83, 1·10)
4000–4099	2·8	1·49 (1·04, 2·13)	2·1	1·03 (0·69, 1·55)
4100–4199	1·9	0·98 (0·57, 1·68)	1·8	0·83 (0·48, 1·46)
4200–4299	2·2	0·98 (0·53, 1·82)	2·9	1·36 (0·82, 2·27)
4300–4399	2·9	1·42 (0·74, 2·70)	2·3	1·03 (0·51, 2·11)
4400–4499	2·6	1·21 (0·48, 3·03)	2·6	1·17 (0·47, 2·88)
4500–4999	3·5	1·70 (0·93, 3·10)	3·2	1·44 (0·78, 2·66)
≥5000	3·0	1·09 (0·13, 9·03)	15·2	7·22 (2·71, 19·26)

a: All estimates were based on two-level logistic regression models. Facilities represent units at level two and individuals within facilities are observations at level one. We adjusted for country, maternal age, marital status, education (total years of school attendance), obesity, diabetes, parity, infant sex, and gestational age.

Additional Table 4 Prevalence and adjusted odds ratios of maternal and perinatal mortality and morbidity by birthweight in singleton term infants born through vaginal deliveries

	Maternal mortality and morbidity		Perinatal mortality and morbidity	
**Birthweight (g)**	**Prevalence (%)**	**Adjusted OR (95% CI)[a]**	**Prevalence (%)**	**Adjusted OR (95% CI)[a]**
**Africa**	**N=** 59453			
3000–3499	3·3	1·00	3·8	1·00
3500–3999	4·0	1·10 (0·97, 1·25)	3·3	0·90 (0·79, 1·01)
4000–4099	5·8	1·44 (1·12, 1·86)	2·9	0·82 (0·61, 1·12)
4100–4199	4·7	1·19 (0·75, 1·89)	2·5	0·81 (0·47, 1·42)
4200–4299	4·6	1·25 (0·78, 2·00)	2·2	0·71 (0·39, 1·29)
4300–4399	5·6	0·99 (0·55, 1·77)	4·0	1·25 (0·69, 2·30)
4400–4499	8·3	2·19 (1·15, 4·16)	2·8	1·14 (0·46, 2·84)
4500–4999	6·7	1·21 (0·79, 1·85)	4·0	1·23 (0·75, 2·01)
≥5000	11·5	2·39 (1·15, 4·96)	16·7	5·49 (3·06, 9·86)
**Asia**	**N=** 65956			
3000–3499	3·3	1·00	1·5	1·00
3500–3999	3·3	1·25 (1·04, 1·49)	1·3	0·94 (0·75, 1·17)
4000–4099	2·2	1·42 (0·69, 2·93)	1·8	1·35 (0·66, 2·77)
4100–4199	1·9	0·66 (0·18, 2·45)	1·9	1·78 (0·65, 4·88)
4200–4299	3·3	2·56 (0·90, 7·24)	2·4	1·76 (0·55, 5·65)
4300–4399	1·4	0·88 (0·11, 7·10)	5·8	4·77 (1·66, 13·73)
4400–4499	3·0	4·12 (0·54, 31·68)	3·0	2·24 (0·29, 17·04)
4500–4999	6·9	3·77 (1·13, 12·61)	6·9	4·51 (1·58, 12·90)
≥5000	22·2	4·72 (1·27, 17·52)	38·9	70·37 (24·41, 202·9)
**Latin America**	**N=** 57778			
3000–3499	2·2	1·00	1·8	1·00
3500–3999	2·3	1·12 (0·97, 1·29)	1·7	0·92 (0·78, 1·08)
4000–4099	2·6	1·41 (0·92, 2·16)	1·7	0·96 (0·58, 1·59)
4100–4199	2·2	1·14 (0·63, 2·06)	1·6	0·92 (0·47, 1·79)
4200–4299	2·1	0·93 (0·43, 1·99)	2·3	1·22 (0·62, 2·40)
4300–4399	2·6	1·29 (0·56, 2·95)	2·1	1·06 (0·43, 2·61)
4400–4499	3·7	1·75 (0·68, 4·5)	3·0	1·48 (0·54, 4·08)
4500–4999	2·9	1·38 (0·59, 3·19)	4·3	2·34 (1·17, 4·65)
≥5000	4·5	2·11 (0·25, 17·97)	18·2	10·30 (3·35, 31·68)

a: All estimates were based on two-level logistic regression models. Facilities represent units at level two and individuals within facilities are observations at level one. We adjusted for country, maternal age, marital status, education (total years of school attendance), obesity, diabetes, parity, infant sex, and gestational age.

Additional Table 5 Prevalence and adjusted odds ratios of maternal and perinatal mortality and morbidity by birthweight in singleton term infants in countries with less than 10% of missing value for BMI

Birthweight	Maternal mortality and morbidity		Perinatal mortality and morbidity	
	**Prevalence (%)**	**Adjusted OR (95% CI)[a]**	**Prevalence (%)**	**Adjusted OR (95% CI)[a]**
**Africa**	**N=36325**			
3000–3499	9·6	1·00	3·5	1·00
3500–3999	10·8	1·10 (1·00, 1·21)	2·7	0·83 (0·70, 0·98)
**4000–4099**	13·9	1·47 (1·21, 1·79)	3·4	0·84 (0·59, 1·19)
**4100–4199**	13·7	1·44 (1·07, 1·94)	2·4	0·82 (0·44, 1·54)
4200–4299	15·5	1·66 (1·26, 2·19)	3·1	0·95 (0·55, 1·63)
4300–4399	18·6	1·62 (1·14, 2·29)	1·9	0·59 (0·23, 1·47)
**4400–4499**	20·6	2·34 (1·54, 3·56)	2·4	0·89 (0·32, 2·44)
4500–4999	18·7	1·81 (1·39, 2·35)	3·6	1·00 (0·59, 1·68)
≥5000	25·6	3·42 (2·14, 5·47)	12·4	3·26 (1·82, 5·86)
**Asia**	**N=91595**			
3000–3499	4·7	1·00	1·7	1·00
3500–3999	5·4	1·38 (1·24, 1·52)	1·6	1·05 (0·9, 1·24)
4000–4099	5·4	1·86 (1·35, 2·56)	2·1	1·49 (0·94, 2·35)
**4100–4199**	6·7	2·25 (1·50, 3·38)	1·2	1·01 (0·45, 2·29)
4200–4299	5·0	2·03 (1·17, 3·52)	4·1	2·80 (1·58, 4·99)
4300–4399	11·5	4·42 (2·60, 7·50)	3·3	2·44 (1·06, 5·64)
4400–4499	7·9	3·81 (1·68, 8·60)	4·0	2·99 (1·07, 8·34)
4500–4999	10·0	3·38 (1·99, 5·74)	6·0	4·12 (2·24, 7·56)
≥5000	17·3	7·51 (3·25, 17·33)	26·9	28·44 (14·81, 54·61)
**Latin America**	**N=49909**			
3000–3499	3·0	1·00	1·5	1·00
3500–3999	3·1	1·05 (0·92, 1·20)	1·4	0·97 (0·80, 1·18)
4000–4099	3·3	1·17 (0·79, 1·72)	1·7	1·19 (0·70, 2·02)
4100–4199	2·3	0·82 (0·44, 1·52)	1·5	1·11 (0·52, 2·37)
4200–4299	3·4	1·14 (0·64, 2·02)	2·6	1·89 (0·99, 3·59)
4300–4399	3·4	1·26 (0·61, 2·60)	0·9	0·68 (0·17, 2·75)
4400–4499	3·4	1·11 (0·44, 2·79)	2·8	2·01 (0·73, 5·53)
**4500–4999**	5·0	1·67 (0·97, 2·88)	4·3	3·15 (1·76, 5·64)
≥5000	12·0	3·82 (1·04, 14·06)	12·0	8·93 (2·60, 30·67)

a: All estimates are based on two-level logistic regression models. Facilities represent units at level two and individuals within facilities are observations at level one. We adjusted for country, maternal age, marital status, education (total years of school attendance), obesity, diabetes, parity, infant sex and gestational age.

Additional Table 6 Prevalence and adjusted odds ratios of maternal and perinatal mortality and morbidity by birthweight centile in singleton term infants in countries with less than 10% of missing value for BMI

**Birthweight centile**	**Maternal mortality and morbidity**		**Perinatal mortality and morbidity**	
	**Prevalence (%)**	**Adjusted OR (95% CI)**[a, c]	**Prevalence (%)**	**Adjusted OR (95% CI)**[a, c]
**Africa**	**N=36325**			
P_{50}–P_{74}	9·5	1·00	3·4	1·00
P_{75}–P_{89}	10·5	1·24 (1·10, 1·40)	2·6	0·85 (0·69, 1·06)
P_{90}–P_{94}	9·6	1·10 (0·93, 1·29)	4·1	1·08 (0·85, 1·38)
P_{95}–P_{96}	10·9	1·46 (1·15, 1·86)	4·1	1·15 (0·80, 1·66)
**≥P_{97}**	13·0	1·70 (1·48, 1·96)	4·4	1·07 (0·85, 1·33)
**Asia**	**N=91595**			
P_{50}–P_{74}	5·0	1·00	1·8	1·00
P_{75}–P_{89}	5·1	1·03 (0·92, 1·15)	2·0	1·02 (0·86, 1·19)
P_{90}–P_{94}	4·8	1·04 (0·89, 1·21)	1·7	0·88 (0·70, 1·11)
P_{95}–P_{96}	5·1	1·20 (0·98, 1·46)	2·4	1·08 (0·83, 1·41)
**≥P_{97}**	6·4	1·41 (1·25, 1·59)	2·5	1·12 (0·94, 1·34)
**Latin America**	**N=49909**			
P_{50}–P_{74}	3·0	1·00	1·3	1·00
P_{75}–P_{89}	3·1	1·06 (0·89, 1·26)	1·5	1·11 (0·86, 1·42)
P_{90}–P_{94}	3·0	1·04 (0·82, 1·32)	1·1	0·82 (0·56, 1·19)
P_{95}–P_{96}	3·1	1·09 (0·78, 1·51)	1·8	1·23 (0·79, 1·90)
**≥P_{97}**	3·3	1·15 (0·93, 1·43)	2·2	1·49 (1·14, 1·96)

a: All estimates are based on two-level logistic regression models. Facilities represent units at level two and individuals within facilities are observations at level one. We adjusted for country, maternal age, marital status, education (total years of school attendance), obesity, diabetes, parity, infant sex, and gestational age.

Additional Table 7 Prevalence and adjusted odds ratios of maternal and perinatal mortality and morbidity by birthweight with exclusion of obesity and diabetes from the regression models in singleton term infants

Birthweight	Maternal mortality and morbidity		Perinatal mortality and morbidity	
	**Prevalence (%)**	**Adjusted OR (95% CI)[a]**	**Prevalence (%)**	**Adjusted OR (95% CI)[a]**
**Africa**	**N=67546**			
3000–3499	6·0	1·00	4·6	1·00
3500–3999	7·2	1·10 (1·01, 1·20)	4·2	0·95 (0·85, 1·05)
**4000–4099**	9·2	1·45 (1·22, 1·73)	4·0	0·98 (0·77, 1·24)
**4100–4199**	10·6	1·51 (1·14, 1·99)	3·4	0·93 (0·60, 1·44)
4200–4299	11·7	1·62 (1·25, 2·11)	3·4	0·87 (0·56, 1·34)
4300–4399	14·3	1·74 (1·26, 2·42)	5·0	1·32 (0·81, 2·14)
**4400–4499**	**15·5**	2·32 (1·55, 3·48)	3·8	1·26 (0·64, 2·51)
4500–4999	15·0	1·89 (1·47, 2·42)	5·4	1·38 (0·95, 1·99)
≥5000	21·2	3·27 (2·08, 5·12)	14·4	4·06 (2·44, 6·73)
**Asia**	**N=91595**			
3000–3499	4·7	1·00	1·7	1·00
3500–3999	5·4	1·38 (1·25, 1·53)	1·6	1·07 (0·91, 1·26)
4000–4099	5·4	1·87 (1·36, 2·58)	2·1	1·55 (0·98, 2·44)
**4100–4199**	6·7	2·26 (1·51, 3·40)	1·2	1·07 (0·47, 2·42)
4200–4299	5·0	2·05 (1·18, 3·56)	4·1	3·06 (1·72, 5·42)
4300–4399	11·5	4·46 (2·63, 7·57)	3·3	2·61 (1·13, 6·03)
4400–4499	7·9	3·86 (1·71, 8·70)	4·0	3·43 (1·24, 9·49)
4500–4999	10·0	3·45 (2·04, 5·85)	6·0	4·52 (2·47, 8·28)
≥5000	17·3	7·52 (3·25, 17·39)	27·0	31·51 (16·52, 60·12)
**Latin America**	**N=87518**			
3000–3499	2·4	1·00	2·1	1·00
3500–3999	2·6	1·14 (1·02, 1·28)	2·0	0·95 (0·84, 1·07)
4000–4099	3·0	1·44 (1·07, 1·93)	2·3	1·08 (0·78, 1·51)
4100–4199	2·7	1·27 (0·86, 1·89)	2·2	0·99 (0·64, 1·52)
4200–4299	2·5	1·11 (0·69, 1·77)	2·8	1·27 (0·81, 1·98)
4300–4399	3·0	1·33 (0·77, 2·30)	2·2	0·94 (0·50, 1·78)
4400–4499	2·9	1·19 (0·57, 2·45)	2·9	1·27 (0·62, 2·59)
**4500–4999**	3·5	1·55 (0·98, 2·47)	4·0	1·88 (1·22, 2·89)
≥5000	5·5	2·39 (0·70, 8·13)	16·4	8·07 (3·84, 16·95)

a: Data are adjusted odds ratio (95% CI). All estimates are based on two-level logistic regression models. Facilities represent units at level two and individuals within facilities are observations at level one. We adjusted for country, maternal age, marital status, education (total years of school attendance, ref ≥ 13 years), parity, infant sex, and gestational age.

Additional Table 8 Prevalence and adjusted odds ratios of maternal and perinatal mortality and morbidity by birthweight centile with exclusion of obesity and diabetes from the regression models in singleton term infants

Birthweight centile	Maternal mortality and morbidity		Perinatal mortality and morbidity	
	Prevalence (%)	Adjusted OR (95% CI)[a]	Prevalence (%)	Adjusted OR (95% CI)[a]
**Africa**	**N=67546**			
P_{50}–P_{74}	6·3	1·00	4·3	1·00
P_{75}–P_{89}	6·6	1·18 (1·05, 1·31)	4·3	0·98 (0·86, 1·12)
P_{90}–P_{94}	5·7	1·09 (0·95, 1·26)	5·0	1·08 (0·92, 1·26)
P_{95}–P_{96}	6·6	1·31 (1·06, 1·62)	5·4	1·14 (0·91, 1·44)
**≥P_{97}**	7·2	1·57 (1·39, 1·78)	5·8	1·11 (0·97, 1·27)
**Asia**	**N=91595**			
P_{50}–P_{74}	5·0	1·00	1·8	1·00
P_{75}–P_{89}	5·1	1·03 (0·92, 1·15)	2·0	1·02 (0·87, 1·20)
P_{90}–P_{94}	4·8	1·04 (0·89, 1·21)	1·7	0·89 (0·71, 1·12)
P_{95}–P_{96}	5·1	1·20 (0·99, 1·46)	2·4	1·10 (0·84, 1·42)
**≥P_{97}**	6·4	1·42 (1·26, 1·61)	2·5	1·16 (0·97, 1·38)
**Latin America**	**N=87518**			
P_{50}–P_{74}	2·4	1·00	2·0	1·00
P_{75}–P_{89}	2·7	1·13 (0·98, 1·31)	2·2	1·08 (0·93, 1·27)
P_{90}–P_{94}	2·6	1·06 (0·87, 1·29)	1·8	0·88 (0·70, 1·11)
P_{95}–P_{96}	2·9	1·21 (0·92, 1·58)	2·1	0·97 (0·71, 1·33)
**≥P_{97}**	3·0	1·24 (1·04, 1·48)	2·8	1·33 (1·11, 1·60)

a: Data are adjusted odds ratio (95% CI). All estimates are based on two-level logistic regression models. Facilities represent units at level two and individuals within facilities are observations at level one. We adjusted for country, maternal age, marital status, education (total years of school attendance, ref ≥ 13 years), parity, infant sex, and gestational age.
